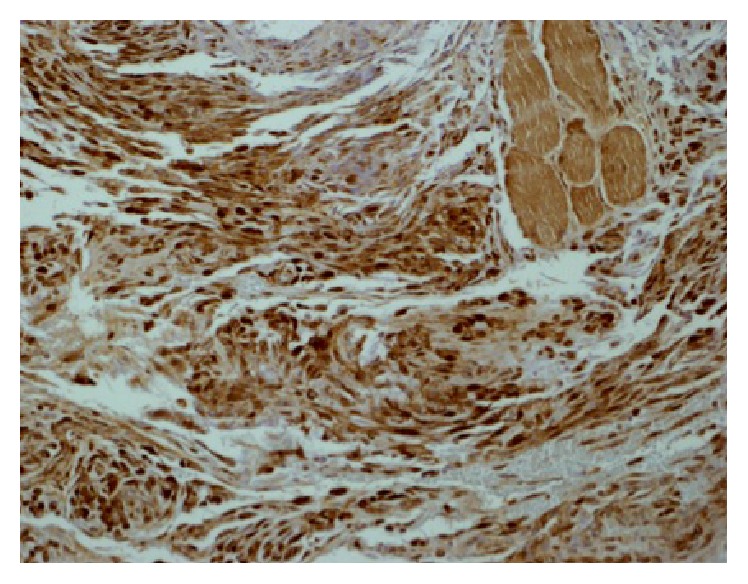# Oral Neurothekeoma of the Right Buccal Mucosa

**DOI:** 10.1155/2016/4709753

**Published:** 2016-09-08

**Authors:** Alex C. Tham, Nandini L. Chilagondanahalli, Manish M. Bundele, Jeevendra Kanagalingam

**Affiliations:** ^1^Department of Otolaryngology, Tan Tock Seng Hospital, Singapore; ^2^Department of Pathology, Tan Tock Seng Hospital, Singapore; ^3^Lee Kong Chian School of Medicine, Singapore

## Abstract

Oral neurothekeoma or nerve sheath myxoma is a rare benign oral tumour of nerve sheath origin. Historically, this tumour has been subclassified as myxoid (classic), mixed, or the cellular type, depending on the amount of myxoid stroma and cellularity. We present a case of oral neurothekeoma (mixed type) of the buccal mucosa. The tumour was completely excised. No recurrence was detected in the last 3 years after local excision.

## 1. Introduction

Oral neurothekeoma or nerve sheath myxoma is a rare benign oral tumour of nerve sheath origin. It was first described in 1969, by Harkin and Reed [1]. This tumour, most commonly, arises within the dermis and subcutaneous tissues on the face and upper extremities. It is extremely rare in the intraoral region [2–4]. We present a case of oral neurothekeoma (mixed type) of the buccal mucosa.

## 2. Case Presentation

A 45-year-old Chinese man presented with a 2-year history of a mass within his right buccal mucosa. This mass had increased in size in the week prior to presentation. He is a nonsmoker and a social drinker, with no significant past medical history.

Physical examination revealed a 2 cm mucosal swelling just posterior to the right oral commissure. There were no palpable cervical lymph nodes.

**Figure 1:**
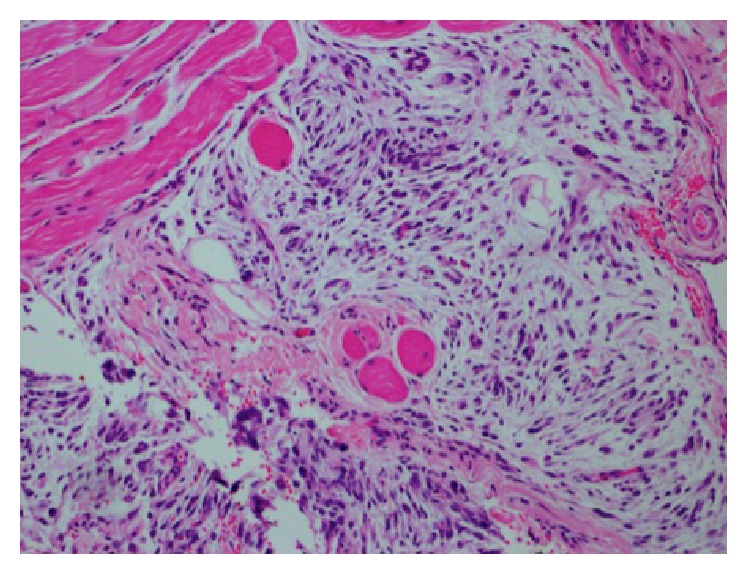


An excisional biopsy of the buccal mass was performed, which revealed features of a myxoid spindle cell proliferation. The lesion was composed of whorls and fascicles of spindle cells demonstrating nuclear hyperchromasia with minimal pleomorphism (Figure 1). The stroma is largely myxoid in nature and no mitosis or necrosis was evident.

Immunohistochemical analyses were positive for S100 protein (Figure 2) but negative for HMB45, Melan A, CD34, cytokeratins (AE1/3, MNF 116), EMA, desmin, myogenin, and smooth muscle actin.

Our patient has been followed up for the last three years. No evidence of recurrence has been detected to date.

## 3. Discussion

Oral neurothekeoma or nerve sheath myxoma is a rare benign oral tumour of nerve sheath origin. Oral neurothekeoma has been reported to occur in locations such as the tongue [4–6], buccal mucosa [3, 7], palate [8], lip [2, 9], retromolar pad [7], and gingiva [10, 11]. Historically, this tumour has been subclassified as myxoid (classic), mixed, or the cellular type, depending on the amount of myxoid stroma and cellularity.

The diagnosis is based on morphological appearances in conjunction with the immunoprofile. S100 positivity is observed in most myxoid cases [3, 10, 12] but can be negative in the cellular variant [11]. Cases with S100 negativity may benefit from stains for NKI/C3, which have shown positivity in the cellular variants [11].

Our case was S100 positive and negative for cytokeratins (excluding spindle cell carcinoma), smooth muscle markers (desmin, myogenin, and smooth muscle actin, excluding a smooth muscle tumour), and melanoma markers (HMB45 and Melan A).

The most important differential diagnosis in S100 positive spindle cell tumour is melanoma with a spindle cell morphology, due to the vastly different prognosis. Absence of significant cellular atypia and mitoses should favour a neurothekeoma. Although HMB45 and Melan A are negative in neurothekeoma, it is known that spindle cell type melanoma can be negative for HMB45 and also Melan A. Hence, immunohistochemistry may not help in arriving at a definitive diagnosis in these S100 positive tumours. In such instances, morphological appearances and clinical correlation are essential. In some cases, careful follow-up with absence of recurrence or metastases may be the only indicator of benignity.

Complete local excision appears to be curative. No recurrence has been reported for oral neurothekeoma, which has been completely excised, thus far. In our follow-up with our patient, no recurrence was detected in the last 3 years after local excision.

In summary, oral neurothekeoma is an uncommon benign soft tissue tumour of peripheral nerve sheath origin. A distinct immunohistochemical profile of this benign tumour remains to be determined. Complete local excision is curative for oral neurothekeomas, with no recurrence reported thus far.

## Figures and Tables

**Figure 2:**